# Angioedema as the first presentation of B-cell non-Hodgkin lymphoma – an unusual case with normal C1 esterase inhibitor level: a case report

**DOI:** 10.1186/1756-0500-7-495

**Published:** 2014-08-07

**Authors:** Sonali Sihindi Chapa Gunatilake, Harith Wimalaratna

**Affiliations:** Registrar in Medicine, Teaching Hospital, Kandy, Sri Lanka; Consultant Physician, Teaching Hospital, Kandy, Sri Lanka

**Keywords:** Acquired angioedema, Lymphoproliferative disorder, C1 esterase inhibitor, C1q level, C4 level, B-cell non-Hodgkin lymphoma

## Abstract

**Background:**

Acquired angioedema is a rare but recognized manifestation of lymphoproliferative disorders due to deficiency in C1 esterase inhibitor. Normal level of C1 esterase inhibitor proteins in association with angioedema due to lymphoproliferative disease is a rare and an uncommon finding caused by antibodies produced from the underlying disease. Antibodies cause inactivation of C1 esterase inhibitor, thus resulting in C1 esterase inhibitor dysfunction despite of normal quantity of C1 esterase inhibitor.

**Case presentation:**

A 50-year-old Sri Lankan male presented with first episode of angioedema without any family history. Physical examination revealed mild pallor with swelling of tongue, lips and perioral region. On investigations, erythrocyte sedimentation rate was persistently high and bone marrow with immunohistochemistry revealed infiltration with B-cell type low grade non-Hodgkin lymphoma. Computed tomography scan of the chest and abdomen showed paratracheal and subcarinal lymphadenopathy and splenomegaly, with the findings being compatible with lymphoma. He had normal C1 esterase inhibitor protein level with reduced activity and low C1q, C4 levels indicating antibodies against C1 esterase inhibitor causing dysfunctional C1 esterase inhibitor.

**Conclusion:**

Adult onset angioedema should prompt physicians to suspect underlying lymphoproliferative disorder despite of C1 esterase inhibitor protein level being normal. Though uncommon, presence of antibodies against C1 esterase inhibitor secondary to lymphoproliferative disorder should be considered in the presence of normal C1 esterase inhibitor protein levels with low functional capacity in the background of acquired angioedema.

## Background

Angioedema is a life-threatening condition described as a transient, non-pruritic, non-pitting localized swelling of cutaneous and mucosal tissues. It often presents with facial, tongue, laryngeal or abdominal edema [1, 2]. Several forms of angioedema are described; hereditary, acquired, allergen associated and idiopathic. Acquired angioedema is reported as a rare clinical manifestation [2–4]. However it is a well-recognized phenomenon associated with non-Hodgkin lymphoma (NHL) [5–7], commonly B cell type [1, 8] as well as other lymphoproliferative disorders (lymphosarcoma, chronic lymphocytic leukemia, Waldenstrom’s macroglobulinemia and multiple myeloma [4, 9]). Angioedema usually precede the diagnosis of lymphoproliferative disease or may occur several years after the diagnosis [5, 10]. Described mechanisms of acquired angioedema are either increased activation resulting consumption of C1 esterase inhibitor leading to reduced levels, commonly seen in lymphoproliferative diseases [11] or antibodies against C1esterase inhibitor as seen in autoimmune diseases. We report a rare case where angioedema was the first presentation of B-cell NHL. The C1 esterase inhibitor (C1-INH) protein levels were normal with decreased level of activity due to antibodies against C1 esterase inhibitor protein causing dysfunctional C1-INH, an uncommon finding in lymphoproliferative diseases.

## Case presentation

A 50-year-old previously well Sri Lankan male patient presented to the Oro-Maxillo-Facial (OMF) surgical unit and then referred to the general medical unit with the complaint of sudden onset swelling of lips, tongue and face for 24 hours. It was painless but progressive over the day. There was no peri-orbital swelling or peripheral edema. He had not experienced dyspnea, wheezing, abdominal pain or body itching. There were no identifiable precipitating factors attributing to angioedema. He denied any history of atopy or allergy. It was the first episode of this nature that he had experienced in his life and there were no previous documented cases of angioedema in his family. He was not on any short or long term medication. On detailed history, patient had experienced loss of appetite and loss of weight (8 kg) over the past 3 months but denied fever, night sweats, chronic cough, joint swelling, oral ulcers and rashes.

On examination, his weight was 49 kg, swelling of the tongue, lips and perioral region were noted. There was mild pallor without icterus but no lymphadenopathy, hepato-splenomegaly or abdominal masses. There were no dental caries, oral ulcers, joint swelling, rashes or bone tenderness. He had a regular pulse with blood pressure of 120/80 mmHg. Respiratory, neurological and loco-motor system examination were unremarkable.

Subsequent investigations revealed hemoglobin of 10.3 g/dL, white cell count of 5.52×10^3^/μL (neutrophils – 38.6%, lymphocytes – 45%, monocytes-14%, eosinophils-1.3% and basophils-0.7%) and a platelet count of 207×10^3^/μL. Blood picture showed normochromic normocytic red cells with marked rouleaux formation, total white cell count was normal with lymphocytic predominance and normal platelets. No atypical cells were seen. Erythrocyte sedimentation rate was 130 mm in 1^st^ hour. Serum lactate dehydrogenase level was 960 IU/L (normal: 100–300 IU/L), C-reactive protein, liver profile and renal profile were normal. Mantoux test was negative. Serology for anti-nuclear antibodies (ANA), retroviral studies, Venereal disease research laboratory (VDRL) test, hepatitis B surface antigen, hepatitis C antibodies, Epstein-Barr virus and cytomegalovirus antibodies were negative.

C1 esterase inhibitor protein level was 23.71 mg/dl (15-35 mg/dL; measured by radio immunediffusion technique) and the functional percentage of C1 esterase inhibitor was 15% (normal >67%, equivocal 41-67%, abnormal <41%) at the time of angioedema. The serum compliment levels were as follows; C3 level – 110 mg/dL (75-165 mg/dL), C4 – 2 mg/dl (14-54 mg/dL) and C1q – 26% (75 -125%).

Bone marrow showed marked hyper cellularity with diffuse infiltration of homogenously mature lymphoid cells (70%). Blasts cells were 1% of marrow nucleated cells. Erythropoiesis, granulopoiesis and megakaryopoiesis were markedly suppressed. Features were compatible with bone marrow infiltration by low grade non-Hodgkin lymphoma (NHL). Immunohistochemistry of the bone marrow revealed CD20 positivity with negative results for CD10, CD3, CD138, CD23 and terminal deoxynucleotidyl transferase (TdT) markers, indicating compatibility with B-cell non-Hodgkin lymphoma. Soluble interleukin 2 receptor levels were not carried out due to unavailability of the laboratory facilities.

**Figure 1:**
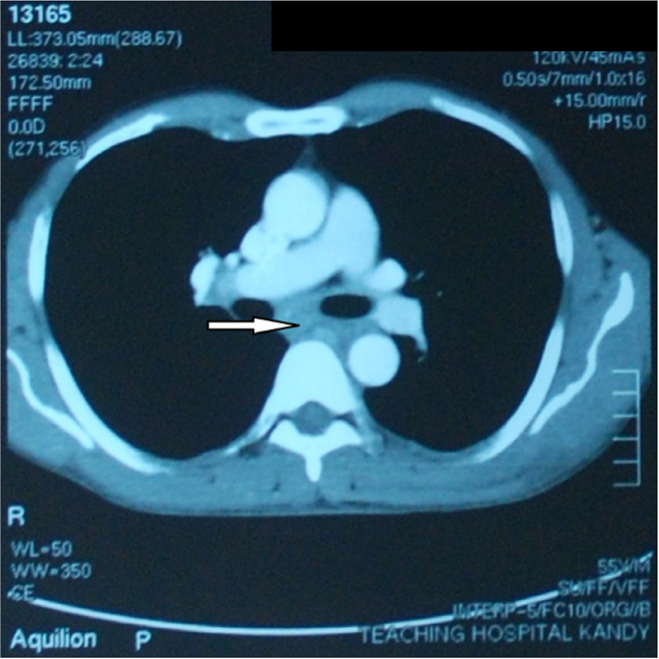
**Contrast enhanced computed tomography scan of chest showing sub-carinal lymphadenopathy.** (Arrow indicates sub-carinal lymphadenopathy).

Electrocardiogram showed sinus rhythm and chest roentgenogram was normal. Ultrasound scan of the abdomen showed mild splenomegaly (15 cm). Contrast enhanced computed tomography (CT) scan of the chest, abdomen and pelvis showed right pretracheal and subcarinal lymphadenopathy with mild splenomegaly compatible with the diagnosis of lymphoma (Figure 1).

Patient was diagnosed to have B cell NHL with an episode of acquired angioedema on presentation. He did not have any life threatening episodes of angioedema but was managed initially with steroids and danazole, to which he responded slowly but completely and started on chemotherapy for the management of NHL. He is on oncology clinic follow-up and he did not develop any further episodes of angioedema thereafter up to 6 months follow-up after starting chemotherapy. Subsequent measurements of complement levels including C4 and C1q were within normal limits.

## Discussion

Angioedema is a life-threatening condition which is being described as a transient, non-pruritic, non-pitting localized swelling of cutaneous and mucosal tissues, which often involves upper respiratory tract or gastrointestinal tract [1, 2]. Neurological and cardiac involvement had also been described [4, 12]. There are several subtypes, (1) Hereditary, (2) Acquired, (3) Allergen associated and (4) Idiopathic [1].

Acquired angioedema shares many features similar to hereditary angioedema but differs by the absence of a positive family history of angioedema and late onset presentation, usually after the age of 40 [1]. It was first described by Caldwell in 1972 [13] and is a rare condition with an estimated prevalence of 1:100 000 to 1:150 000 [14]. Pathophysiology reveals that when the C1 esterase inhibitor protein level is decreased due to excessive consumption, or dysfunctional due to the presence of antibodies against C1-INH (although C1-INH protein level is normal), inhibition of the complements and the kinin system is defective. This results in an abnormal exaggerated response in the compliment pathway causing increased vascular permeability (by mediators such as bradykinin) causing angioedema. Two types of acquired angioedema is described in the literature.

Type I - Low level of C1-INH (<30% of normal); seen in lymphoproliferative disease or paraneoplastic syndromes.

Type II - Antibodies against C1-INH present; seen in connective tissue disease.

Acquired angioedema (AAE) is a rare phenomenon in association with lymphoproliferative disorders. Associated lymphoproliferative disorders range from monoclonal gammopathy of unknown significance –MGUS (35%) to non-Hodgkin lymphoma (20%) [1, 10]. B-cell non-Hodgkin lymphoma is the commonest of lymphoma to give rise to angioedema, especially recurrent angioedema [1, 5–8], and angioedema may precede or follow the diagnosis of NHL [5, 10, 15]. Few cases are reported with acquired angioedema being the first manifestation of NHL [5].

Pathogenesis of AAE in B-cell NHL is incompletely understood [16] but suggests activation of classical complement pathway from immune complexes or monoclonal antibodies produced by the tumor tissue leading to consumption of C1-INH. Thus serum C1, C1q, C2, C4 level and C1-INH protein levels will be lower than the normal (AAE Type I). This mechanism is well described and reported in many case reports [2–4, 6, 8, 17]. Although not common, a monoclonal antibody that inactivates the C1-INH leading to non-functional C1-INH resulting in angioedema had also been reported [15, 17, 18]. Normally C1-INH is cleaved by the targeted proteases. Cleaved products bind to the proteases in the complement and kinin pathways irreversibly and render them inactive. In the presence of inhibitor antibodies to C1-INH, still C1-INH is cleaved but cannot bind to the proteases to make them inactive, thus the proteases continue to function. This results in uncontrolled activation of complement pathway and production of bradykinin leading to angioedema. It gives rise to normal C1-INH protein levels with a defective function and low C1q and C4 levels (as a result of activation of classical complement pathway and excessive consumption of the compliment components) as well as antibodies against C1-INH can be identified (produced by the lymphoma cells). This mechanism (AAE Type II) is well described in relation to connective tissue diseases causing acquired angioedema, but less commonly with lymphoproliferative diseases. Gaur et al. had reported a case with lymphoma associated angioedema with normal C1-INH and C4 levels, in which the mechanism was not well understood.

The patient in this case report was diagnosed to have B-cell NHL following investigations for a single episode of acquired angioedema, which is a rare presentation. The underlying cause for an acquired angioedema should be aggressively pursued in such a patient to exclude an underlying lymphoproliferative disease. He had low C1q and C4 levels, indicating an acquired form of angioedema with normal C1-INH protein levels and low functional C1-INH (due to antibody mediated defective function of C1-INH), which is less commonly described in relation to lymphoproliferative disorders in literature. The antibodies against C1-INH and CH50 level were not carried out due to lack of laboratory facilities but low levels of C4 and functional C1-INH irrespective of normal level of C1-INH proteins supported the diagnosis.

## Conclusion

This rare case of acquired angioedema as the first manifestation of non-Hodgkin lymphoma highlights the importance of having a high degree of suspicion to exclude occult malignancies or underlying disease in patients with adult onset angioedema. Although rare, the possibility of auto antibodies inactivating C1-INH (acquired angioedema type II) should be appreciated in the presence of lymphoma, resulting in acquired angioedema with normal C1 esterase protein levels.

## Consent

Written informed consent was obtained from the patient for publication of this case report and any accompanying images. A copy of the written consent is available for review by the Editor-in-chief of this journal.

## Authors’ information

HW (MBBS, MD, FRCP (Edin), FRCP (Lond), FCCP) is a Consultant Physician, Teaching Hospital, Kandy, Sri Lanka. SSCG (MBBS) is a Registrar in Medicine attached to the Teaching Hospital, Kandy, Sri Lanka.

## Abbreviations

AAE: Acquired angioedema
ANA: Anti nuclear antibodies
CT: Computed tomography
C1-INH: C1 esterase inhibitor
MGUS: Monoclonal gammopathy of unknown significance
NHL: Non-Hodgkin lymphoma
OMF: Oro-maxillo-facial
TdT: Terminal deoxynucleotidyl transferase
VDRL: Venereal Disease Research Laboratory.
